# Evaluation of magnetic resonance imaging parameters and compliance with guidelines in soft tissue sarcomas

**DOI:** 10.1186/s12885-026-15569-3

**Published:** 2026-01-13

**Authors:** Madelaine Hettler, Josephine Kirschstein, Isabelle Ayx, Melissa Harbrücker, Franka Menge, Christoph Reißfelder, Stefan Schönberg, Dominik Nörenberg, Matthias F Froelich, Jens Jakob

**Affiliations:** 1 Department of Surgery, Sarcoma Unit, University Medical Center Mannheim, Medical Faculty Mannheim of Heidelberg University, Mannheim, Germany; 2 Department of Radiology and Nuclear Medicine, University Medical Center Mannheim, Medical Faculty Mannheim of Heidelberg University, Mannheim, Germany; 3 DKFZ-Hector Cancer Institute, University Medical Center Mannheim, Heidelberg, Germany; 4 Department of Surgery, University Medical Center Mannheim, Medical Faculty Mannheim of Heidelberg University, Mannheim, Germany

**Keywords:** Soft tissue sarcoma, Magnetic Resonance Imaging, Diagnostic imaging, Multimodal treatment

## Abstract

**Background:**

Accurate imaging plays a crucial role for prognostic assessment and treatment allocation in soft tissue sarcoma (STS). Guidelines recommend contrast-enhanced magnetic resonance imaging (MRI) including diffusion-weighted imaging (DWI) for tumor assessment. This study aims to evaluate the extent to which the MRI protocols used in clinical practice align with the sequences specified in the guideline in the diagnostic work-up of sarcoma patients.

**Methods:**

We analyzed a cohort of patients treated at the Sarcoma Center of the University Medical Center Mannheim (UMM). Pretherapeutic MRI protocols were compared with the recommendations of the German sarcoma guideline.

**Results:**

We analyzed 64 MRI examinations of patients with STS of the extremities and trunk, notably 62 performed at external radiology centers and 2 at UMM.

A pretherapeutic contrast-enhanced MRI was available in 51 of 64 cases (79.7%), of which 40 referrals explicitly indicated a soft tissue tumor. In 3.1% of the cases, the complete set of guideline-recommended sequences was not performed. At least two of the required sequences were consistently available. The most frequently absent sequences were the T1-weighted sequence without fat saturation acquired before and after contrast administration using identical acquisition parameters (82.8%) and DWI sequences (78.1%).

**Conclusions:**

MRI protocol selection is primarily guided by the clinical referral question. Our analysis indicates that a substantial proportion of examinations deviated from recommended protocols. Contributing factors may include inaccurate clinical referral questions, limited guideline awareness, reimbursement constraints in outpatient settings, or diagnostic considerations. Standardized imaging represents a key instrument for quality assurance in the diagnosis of STS and forms the foundation for clinical and translational studies. In the future, the recommendations should be re-evaluated and published in a clear, accessible format to ensure broad implementation.

## Introduction

Soft tissue sarcomas (STS) are a rare and heterogeneous group of malignant mesenchymal tumors characterized by pronounced inter- and intratumoral heterogeneity. More than 100 distinct histological subtypes have been described, with liposarcomas and leiomyosarcomas among the most common [1]. STS can arise in a wide range of anatomical locations; approximately half of the cases occur in the extremities, followed by the trunk, retroperitoneum, and head and neck region. The systematic analysis of European cancer registries revealed an overall annual incidence of STS in Europe of 4.7 per 100,000 inhabitants [2]. 

Histopathological grading based on biopsy specimens currently represents the gold standard for assessing tumor aggressiveness [3]. Low-grade sarcomas are generally managed with primary surgical resection, while high-grade sarcomas require the evaluation of neoadjuvant strategies such as radiation and/or chemotherapy. Accurate diagnostic is fundamental for effective treatment planning and prognosis in patients with STS. Since grading, tumor size and histological subtype have an impact on prognosis [4]. However, due to the pronounced heterogeneity and large volume in up to 68% of STS cases, non-representative biopsies result in under-grading, which may cause inadequate multimodal treatment allocation [5]. In contrast, imaging may has the potential to determine both the dignity and the grading by assessing the entire tumor volume of uncertain soft tissue masses [6, 7]. A prerequisite for this approach is the availability of comprehensive imaging data of sufficient quality, including standardized functional acquisitions, to allow for reliable comparability. Accordingly, the implementation of standardized imaging protocols is essential.

In Germany, the most important guideline for the diagnosis and treatment of STS is the S3 Guideline “Adult Soft Tissue Sarcomas” [8]. This guideline was developed under the auspices of the German Association of the Scientific Medical Societies (Arbeitsgemeinschaft der Wissenschaftlichen Medizinischen Fachgesellschaften, AWMF), following their methodological standards, including a systematic literature review and a Delphi process involving delegates from the respective medical societies. Participating societies included, among others, the German Society for General and Visceral Surgery (DGAV), the Working Group on Imaging in Oncology within the German Cancer Society (ABO/DKG), the German Radiological Society (DRG), and the German Interdisciplinary Sarcoma Group (GISG). As in the European and international guidelines, contrast-enhanced magnetic resonance imaging (MRI) is defined as the imaging modality of choice in cases of suspected sarcoma [8–10]. 

Although CT and PET/CT are employed for the radiological evaluation of retroperitoneal sarcomas, magnetic resonance imaging has long been regarded as the modality of choice for local staging of STS. It enables detailed evaluation of tumor size, margin characteristics, intratumoral signal intensity and heterogeneity, peritumoral edema, and neurovascular or osseous involvement, both at initial diagnosis and during follow-up.

The German guideline’s background text further specifies the recommended MRI protocol (Table 1), including T1- and T2-weighted sequences before and after contrast administration, as well as diffusion-weighted imaging (DWI) in accordance with the recommendations of the European Society of Musculoskeletal Radiology (ESSR) [11]. 

**Table 1:** MRI protocol recommendations according to the German S3 guideline “Adult soft tissue Sarcomas”

1	T1-weighted sequence (spin echo (SE) or turbo spin echo (TSE)) in a long axis orientation (coronal or sagittal)
	T2-weighted sequences
2	• axial plane
3	• long axis
	T1-weighted sequences after intravenous contrast administration
4	• axial plane
5	• long axis
6*	T1-weighted sequence without fat saturation, acquired before and after contrast administration using identical acquisition parameters
7**	Axial T2-weighted sequence without fat saturation A DIXON sequence with adequate spatial resolution is accepted as an alternative
8	Diffusion weighted imaging

* This may have been covered by sequences 1 and 5. In such cases, we considered the MRI set complete

** This may have been covered by sequence 2. In such cases, we considered the MRI set complete

Outside of guideline recommendations, there is no evidence-based, widely accepted or published consensus on a standardized MRI protocol for STS imaging. As a result, multicenter cohort studies to validate parameters for radiologic grading are considerably limited. In clinical practice, patients typically undergo outpatient, non-standardized MRI prior to referral to a specialized sarcoma center for further diagnostic workup and treatment planning.

The aim of this study is to compare the MRI protocols used in routine clinical practice for patients with STS to the recommendations outlined in the German S3 guideline. By analyzing the actual radiological protocols applied, this study seeks to evaluate current imaging practices and identify potential gaps in adherence to guideline-based diagnostic standards.

## Methods

### Study design and patient population

This single-center, non-interventional, retrospective, study was conducted at the University Medical Center Mannheim, Medical Faculty of the University of Heidelberg, Mannheim, Germany. Ethical approval was obtained from the Ethics Committee II of the University of Heidelberg (approval number 2023 − 852). Written informed consent for participation was obtained from all participants. The study was conducted in compliance with the ethical principles of the Declaration of Helsinki and its later amendments.

All patients were treated at the Sarcoma Center Mannheim and selected according to the following inclusion criteria: age ≥ 18 years, histologically confirmed primary STS of the extremities or trunk, and availability of pre-therapeutic MRI imaging. Retroperitoneal sarcomas were excluded from the analysis, as CT imaging is defined as the equivalent imaging modality in the guideline. Therefore, the presence of MRI imaging in these cases cannot be considered as a qualitative criterion.

### Definition of standard MRI protocol

For this study, the recommendations of the German S3 guideline “Adult Soft Tissue Sarcomas,” version 1.1 (AWMF registry number: 032/044OL) were applied. In Chap. 4.1.1 “Imaging“, contrast-enhanced MRI is recommended as the primary imaging modality of choice when a malignant soft tissue tumor is suspected (recommendation 4.1). The in-plane spatial resolution should be approximately 0.5 × 0.5 mm, with a slice thickness of 3 to 5 mm. The acquisition parameters defined as standard imaging within the guideline are presented in Table 1.

### Data analysis

Pre-therapeutic MRI scans were utilized for the analysis, defined as imaging acquired prior to histopathological confirmation of STS diagnosis. MRI sequences were initially identified based on the written radiological reports, when available, and subsequently verified through direct image review. In instances where no written report was accessible, sequences were determined by direct evaluation of the imaging data. All available MRI sequences were included in the analysis.

The acquired sequences were compared to the standard MRI protocol defined in the German S3-guideline with respect to their imaging acquisition parameters.

MRI data were independently reviewed by a medical student (JK) and a surgical resident (MH), and further validated by an experienced radiologist (IA). Image analysis was performed using the syngo.share view diagnostic viewer (VA30, 11250008, 2022, Siemens Healthcare GmbH, Erlangen, Germany).

### Statistical analysis

Data are presented descriptively. Continuous variables will be described by sample size, mean, median, as well as the minimum and maximum values. Discrete variables will be presented as frequencies and percentages. Statistical analyses were performed using Jamovi (version 2.6.26.0).

## Results

### Patient characteristics

A total of 99 patients with available MRI imaging, who were treated at the Sarcoma Center of the University Medical Center Mannheim between 2021 and 2024, were identified. Of these, 35 were excluded because the imaging had been performed after histological diagnosis, e.g. after whoops surgery, leaving 64 cases eligible for analysis.

The analyzed cohort comprised 40 men and 24 women, with a median age of 68 years. The median tumor size was 7 cm. The majority of the analyzed tumors were located in the lower (*n* = 45) and upper (*n* = 7) extremities, followed by the trunk (*n* = 12). Overall, 17 different histological subtypes were represented, the most common being dedifferentiated liposarcoma (*n* = 18) and myxofibrosarcoma (*n* = 8). According to FNCLCC criteria, there were 19 grade (G) 1, 18 G2, and 21 G3 sarcomas, resulting in a balanced distribution. An overview of patient and tumor characteristics is provided in Table 2.

**Table 2:** Patient and tumor characteristics

		*n* or Median (range)
Patient characteristics	Number of patients	64
	Age (in years)	68 (21–99)
	Sex: Female**/**Male	24/40
Tumor characteristics	Primary tumor	64
	Recurrent tumor	0
	Tumor size (in cm)	7 (2 − 25)
Tumor localization	Upper Extremity	7
	Lower Extremity	45
	Trunk	12
Histologic subtype	Well-differentiated liposarcoma	7
	Dedifferentiated liposarcoma	11
	Myxoid liposarcoma	7
	Leiomyosarcoma	7
	Myxofibrosarcoma	8
	Synovial sarcoma	3
	Solitary fibrous tumor	2
	Angiosarcoma	2
	Undifferentiated pleomorphic sarcoma	8
	Other	9
Grading (FNCLCC)	Grade 1	19
	Grade 2	18
	Grade 3	21
	Grade x	6

### Imaging characteristics

A total of 64 MRI examinations of patients with STS of the extremities and trunk were evaluated. Of these, 62 examinations were performed at external radiology centers, and two were conducted at the Department of Radiology, University Medical Center Mannheim. Across all examinations, the median number of performed sequences was 8, with a range of 4 to 26.

In 46/64 cases (71.7%), the explicit clinical question concerned the presence of a soft tissue tumor. In six cases, MRI was indicated for other reasons, including muscle fiber tear (*n* = 1), hematoma (*n* = 2), reflex sympathetic dystrophy (*n* = 1), suspected osteoarthritis (*n* = 1) and suspected meniscus injury (*n* = 1). For 12 patients, the exact clinical question could not be determined.

### Imaging analysis

#### Sequences

**Fig. 1:**
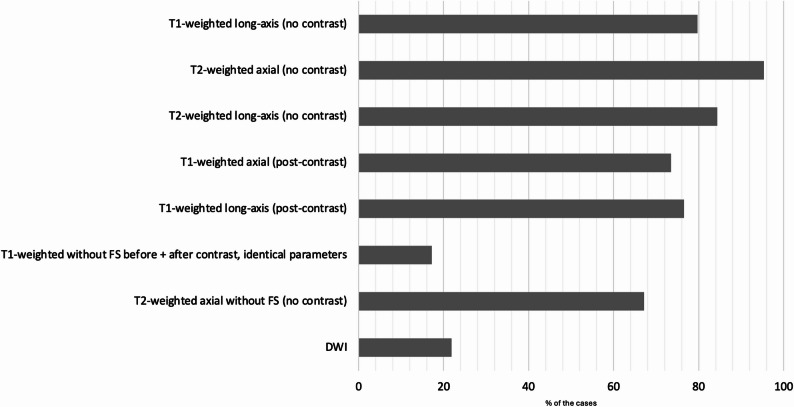
Frequency of guideline-specified MRI sequences. The diagram illustrates the percentage of cases in which the sequences recommended by the S3 guideline were actually performed (n = 64). A complete MRI dataset was available in only 2 out of 64 cases (3.1%)

The guideline recommends the inclusion of at least six specific MRI sequences for STS assessment. The distribution of the sequences in this cohort is summarized in Fig. 1.

Complete guideline-concordant imaging, including all sequences recommended by the guideline, was available in only 2 out of 64 patients (3.1%). In both cases, the examinations were conducted under the suspicion of a tumor, and the diagnoses were confirmed as dedifferentiated liposarcoma of the pelvis in one patient and dedifferentiated liposarcoma of the lower extremity in the other.

In the remaining cases, at least two of the required sequences were consistently available. Missing sequences were distributed as follows: one sequence missing in 12 examinations, two sequences in 11 examinations, three sequences in 18 examinations, four sequences in 9 examinations, five sequences in 11 examinations, and all six sequences missing in one examination.

**Fig. 2:**
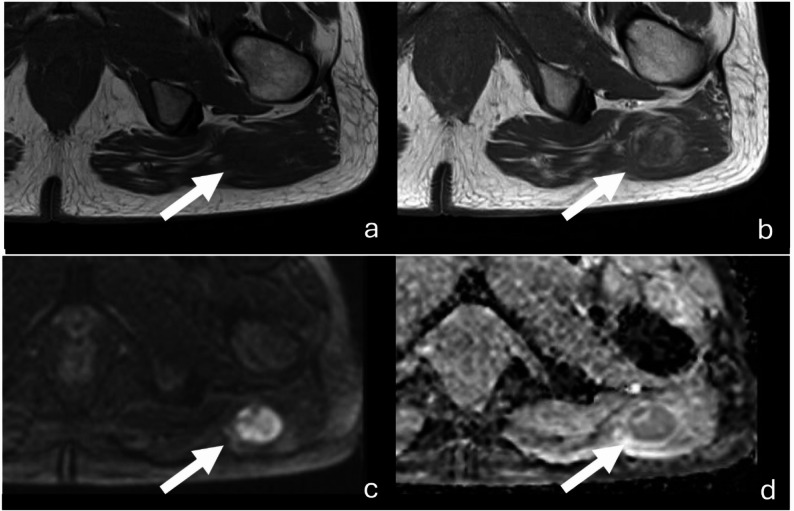
MRI of a myofibroblastic sarcoma (white arrow), G1, of the lower extremity. The figure illustrates the two guideline-recommended MRI sequences most frequently omitted in clinical practice: a T1-weighted sequence, axial without fat saturation, acquired before and after contrast administration using identical acquisition parameters (**a**, **b**), and DWI (**c**) with ADC map (**c**), axial with b-value 800 s/mm² (**d**). Due to the identical parameters in images a and b, contrast enhancement can be directly quantified. In DWI imaging (**c**), diffusion restriction can be confirmed using the corresponding ADC map (**d**)

The most frequently absent sequence was the T1-weighted sequence without fat saturation acquired before and after contrast administration using identical acquisition parameters (*n* = 53; 82.8%). The main reason for the absence of this sequence was the application of fat saturation (*n* = 16 of 45). Diffusion-weighted imaging (*n* = 50; 78.1%) was the second most frequently missed sequence in the analyzed cohort (Fig. 2).

At least one T1-weighted sequence in a long-axis orientation (coronal or sagittal) was available in 51 patients (79.7%). T2-weighted imaging without contrast was performed in 61 patients (95.3%) in the axial plane and in 54 patients (84.4%) in a long-axis orientation. After intravenous contrast administration, T1-weighted sequences were acquired in the axial plane in 47 patients (73.4%) and in a long-axis orientation in 49 patients (76.6%). A T1-weighted sequence without fat saturation before and after contrast application using identical acquisition parameters, as required by the guideline, was available in 11 patients (17.2%). An axial T2-weighted sequence without fat saturation or a Dixon sequence with adequate spatial resolution as an alternative was performed in 43 patients (67.2%).

DWI was included in 14/64 MRI examinations (21.9%). Within this cohort, 12 cases were performed with the clinical question of a tumor, corresponding to a proportion of 26.1% (12/46). In the group without an initial suspicion of tumor, 2 out of 7 patients (28.6%) underwent imaging that included DWI sequences. Analysis of the applied b-values showed that values between 0 and 1000 were used, with b-values of 50, 400, and 800 s/mm² being the most frequently applied. 

#### Contrast enhancement

51/64 patients (79.7%) underwent pre-therapeutic MRI with intravenous contrast administration. In the remaining 13/64 patients (20.3%) without contrast-enhanced imaging, there was no documentation of contraindications (severe allergic reaction or intolerance, severe acute renal failure, or acute dialysis dependency). Among the cases with suspected tumor in the initial radiological work-up, a pre-therapeutic contrast-enhanced MRI was available in 40 of 46 patients (86.9%). In the cohort with a clinical indication other than a suspected mass 5 of 6 patients (83.3%) and in the group with an unknown clinical indication 6 of 12 patients (50%), respectively, underwent contrast-enhanced MRI.

## Discussion

The primary objectives of diagnostic MRI in STS are the assessment of tumor extent and tissue characteristics. Imaging allows the evaluation of tissue density, intratumoral heterogeneity, including necrosis and perfusion as well as tumor margins. From a surgical perspective, MRI also plays a pivotal role in biopsy planning and the assessment of resectability by analyzing the relationship to adjacent critical structures.

Our study demonstrates that in the majority of examined cases, primarily obtained at non-academic outpatient centers, not all sequences for local STS staging recommended by the S3 guideline were included. Although a median of eight sequences per examination was performed, only a very small proportion of cases fulfilled the complete S3 guideline requirements. Most frequently, the T1-weighted sequence without fat saturation acquired before and after contrast administration using identical acquisition parameters, as well as DWI, were not part of the applied imaging protocols. Nevertheless, at least two of the required sequences were consistently available. In the cases analyzed, this approach enabled sufficient anatomical assessment to inform subsequent therapeutic planning. However, for functional evaluation – such as delineating infiltration of critical structures – or for research applications, a higher degree of standardization and comparability of imaging protocols is required. One of the key obstacles to uniform protocol implementation is the relatively low level of awareness and familiarity with the sarcoma imaging recommendations among radiologists outside specialized centers. The S3-guideline itself is extensive and the specific protocol information is embedded deeply within its main text rather than highlighted as succinct, accessible recommendations. As sarcoma is rare, it is questionable whether external radiologists can realistically obtain and assimilate these details during routine practice, which may partially account for the observed protocol deviations. This underlines the need for targeted education initiatives and more user-friendly dissemination of such protocols to the broader radiology community, in conjunction with structured feedback mechanisms [12]. Moreover, the selection of the MRI protocol is primarily guided by the clinical question specified in the referral, which in some cases is imprecisely defined. In the analyzed cohort, a suspected soft tissue tumor was documented in approximately 70% of referrals.

In a large-scale analysis from the USA comprising nearly 400 patients with STS, it was shown that up to 20% of patients did not undergo adequate preoperative imaging. The absence of appropriate imaging was identified as a relevant factor influencing surgical outcomes. The proportion of patients who had received cross-sectional imaging differed significantly between the groups: 92% in the cohort with complete (R0) resections versus only 65% in the cohort with incomplete resections [13]. In this study, “adequate imaging” was defined as CT, MRI, or PET, without specification of a minimal standard. This is likely attributable to the fact that both the American and British guidelines designate imaging as the gold standard for diagnostic workup in STS, yet do not define a precise protocol or minimum requirements [9, 10]. 

Since 2021, the German S3 guideline on “Adult Soft Tissue Sarcomas” has been available, defining a total of at least six different MRI sequences for adequate characterization of STS.

The guideline represents the highest methodological level and is grounded in a systematic review of the evidence, complemented by formal consensus-building processes. Accordingly, it is rigorously researched, methodically coordinated, and transparently published, thereby constituting a binding recommendation for clinical practice. Its primary advantage lies in the establishment of a standardized approach to patient management. Since 2015, consensus guidelines from the ESSR on the imaging of soft tissue tumors have been available as-well. Overall, the criteria cited do not differ from those of the German S3 guideline. But many European recommendations are nonspecific – for example, “Recommended in most cases” or “may also be useful“ – and only the a fluid-sensitive, fat-saturated T1-weighted sequence, is explicitly mentioned, which does not constitute a full MRI protocol [11]. Consequently, a parallel assessment of compliance with ESSR recommendations in this study was not feasible. The 2023 und 2024 guideline updates similarly lack an exact protocol; however, the underlying validated Delphi process of 46 specialized musculoskeletal radiologists from 12 European countries is detailed, and the agreed aspects, e.g. intravenous gadolinium contrast administration (consensus 100%), largely confirm the German S3 recommendations [12, 14]. 

In the diagnosis of STS, MRI serves as the foundation for optimal treatment allocation. Beyond anatomical tumor characterization, contrast-enhanced imaging and DWI are required to identify high-risk tumors associated with an elevated likelihood of local recurrence or metastasis [15]. In such cases, patients may benefit from additional therapies, such as radiotherapy and/or chemotherapy, which can substantially improve prognosis [16]. 

Current ESSR and German S3 guidelines for adult STS primarily recommend conventional MRI protocols focusing on high‑resolution T1‑ and fluid‑sensitive fat‑suppressed T2‑weighted imaging with static post‑contrast sequences for diagnosis, staging, and surveillance. While these standards ensure robust anatomical characterization and are widely implemented in clinical practice, they do not yet fully reflect the capabilities of modern multiparametric MRI, particularly for grading, response assessment and early recurrence detection. In contrast, several expert centers and recent consensus documents now advocate routine integration of perfusion‑weighted/dynamic contrast‑enhanced MRI (PWI/DCE), DWI, Dixon‑based fat–water imaging, and susceptibility‑weighted imaging (SWI) into STS protocols. DCE‑MRI and DWI have repeatedly shown superior performance over size‑based response evaluation criteria in solid tumors (RECIST) for differentiating benign from malignant lesions, characterizing pseudoprogression, and detecting early local recurrence, with quantitative parameters such as K_trans_ (volume transfer constant), K_ep_ (rate constant), iAUC (initial area under the curve), and ADC correlating with cellularity, microvessel density, and Ki‑67 proliferation index [17–19]. 

The French working group led by Crombé et al., identified three dynamic contrast enhanced MRI-based grading criteria to differentiate between low- and high-grade STS: Peritumoral contrast enhancement, the presence of intratumoral necrosis and a pronounced heterogeneous signal intensity [20]. Based on this and other retrospective, small-scale studies, recommendations for a standard MRI protocol for STS have been formulated. Crombé et al. propose a protocol for pretherapeutic imaging in STS that also includes the use of contrast agents and the acquisition of T1- and T2-weighted sequences in multiple planes [21]. K_trans_ and K_ep_ values from DCE-MRI are significantly higher in malignant compared to benign lesions, and correlate with microvessel density and Ki-67 labeling index, supporting their use in grading and biological behavior assessment [22]. Time-intensity curve (TIC) patterns from DCE-MRI are also validated: malignant curves (TIC III, IV) are found in 74% of sarcomas versus 26.5% of benign lesions [23]. Optionally, DWI is included with b-values of 0, 400, and 800–1000 s/mm². Several smaller studies showed that the ADC derived from diffusion-weighted imaging correlated with grading according to FNCLCC and could potentially contribute to tumor characterization as a radiological grading parameter [24, 25]. For this reason, DWI is already firmly established in the German S3 guideline. In the updated ESSR guideline, the recommendation for DWI—with two to three b-values ranging from 50 to 800–1000 s/mm²— also achieved a consensus of 95%, thus aligning with the recommendations of the German S3 guideline [12]. However, DWI is among the sequences most frequently missing in the analyzed cohort. Dixon fat imaging improves characterization of lipomatous tumors and identification of dedifferentiated components, whereas SWI is particularly valuable for visualizing treatment‑induced hemorrhage and necrosis in desmoid‑type fibromatosis, UPS, and other highly cellular sarcomas. Thus, multiparametric MRI approaches similar to those long established in neuro-oncology (e.g., in glioblastoma) are now increasingly applied to STS and consistently demonstrate higher sensitivity for distinguishing responders from non‑responders than conventional imaging alone [26]. The updated 2024 ESSR guidelines explicitly recommend multiparametric MRI for post-neoadjuvant therapy assessment and surveillance, with imaging intervals stratified by tumor grade and type. The American College of Radiology also endorses MRI with and without contrast as the mainstay for local recurrence surveillance, noting the added value of functional imaging [14, 27]. In summary, while ESSR and German S3 standards provide a solid framework for STS imaging, integration of advanced MRI sequences (PWI/DCE, Dixon Fat, SWI) is essential for optimal tumor biology assessment, treatment response evaluation, and recurrence detection. These techniques should be incorporated into future guideline updates to reflect the evolving evidence base and improve patient outcomes.

A significant barrier to S3-guideline compliance, especially in the outpatient setting, is economic: while the German EBM (uniform value scale for reimbursement) requires at least four sequences for extremity MRI, it does not compensate for additional or technically advanced sequences, such as DWI or extensive multi-planar protocols. Consequently, comprehensive guideline-adherent MRI protocols may be economically unfeasible for many radiologists in daily practice, effectively putting high-quality diagnostic standards at odds with financial sustainability [28]. Therefore, realistic minimum standards and streamlined protocols may be necessary to reconcile quality care with economic realities.

Recent advances in deep learning-based protocol acceleration offer a promising solution. These techniques can reduce scan times by up to 70–80% compared to conventional protocols without compromising image quality or diagnostic confidence, thereby enabling the inclusion of essential sequences (like DWI) within economically viable examination slots [29]. Clinical studies have shown that artificial intelligence (AI)-based reconstructions maintain or even improve image sharpness and reduce artifacts, while their rapid acquisition increases patient throughput and overall economic efficiency [30]. The adoption of such protocol acceleration techniques can thus help to bridge the gap between financial constraints and guideline-driven imaging quality.

Standardized MRI imaging provides comparability and constitutes a key element of quality assurance in the diagnosis, treatment and follow-up of STS across centers [31]. 

In contrast to the initial diagnostic setting, follow-up examinations—where the histological subtype is already known—offer the opportunity to tailor imaging protocols to the underlying histology, which can be pragmatically grouped into four MRI imaging clusters. The latest clinical studies and consensus guidelines validate the use of advanced MRI sequences for tailored imaging protocols in STS clusters as follows: (i) Cellular sarcomas (e.g., undifferentiated pleomorphic sarcoma, leiomyosarcoma, rhabdomyosarcoma, malignant peripheral nerve sheath tumor, synovial sarcoma) frequently show intralesional hemorrhage and necrosis, for which SWI and DCE-MRI improve detection of hemorrhage, perfusion abnormalities, and early treatment response, whereas DWI may be partially limited by T2 effects but still allows ADC‑based assessment of cellularity [32]. (ii) Lipomatous tumors (e.g., well‑differentiated and dedifferentiated liposarcoma) are best characterized on T1‑weighted and Dixon‑based fat-water sequences, which delineate fatty versus non‑fatty components and help detect dedifferentiation, while DCE‑MRI and DWI add value in differentiating benign from malignant lipomatous lesions and in grading. Especially DCE-MRI parameters such as K_trans_ and TIC have shown significant differences between sarcomas and benign lesions, supporting their use in this cluster [23]. (iii) Myxoid and chondroid tumors typically demonstrate very high T2 signal and elevated ADC values due to abundant myxoid matrix, tend to extend along fascial or aponeurotic planes, and benefit from DWI and DCE‑MRI for accurate assessment of tumor extent and therapy response [23, 33]. (iv) Fibrous tumors, such as desmoid‑type fibromatosis, usually show low to intermediate T2 signal with variable enhancement, where T2‑weighted imaging is central for response evaluation, and adjunct DWI/DCE‑MRI can help distinguish more cellular from collagen‑rich components [33]. 

In the primary diagnostic work-up of a soft-tissue tumor without prior knowledge of its histology, it is essential to employ an imaging technique that enables adequate depiction of the majority of tumor subgroups. The imaging is essential for optimal surgical decision-making, as it enables assessment of tumor extent, involvement of adjacent structures, and margin status. Careful assessment of tumor margins is a critical determinant of resection extent and can strongly influence both postoperative function, as well as the risk of local recurrence [34]. Complete tumor resection represents one of the most important prognostic factors for the development of local recurrence. Specific imaging features, such as the “tail sign,” may indicate infiltrative growth or pseudocapsule transgression. In these cases, DWI has the potential to differentiate between actual tumor infiltration and reactive edema [35]. Case discussions in multidisciplinary tumor boards and surgery at specialized sarcoma centers have been shown to significantly improve patient outcomes, underlining the importance of precise imaging-based planning [36, 37]. 

A standardized MRI protocol also enables further evaluations, such as radiological grading based on DWI sequences, within the context of clinical studies [24, 25]. Radiological grading may provide a more representative assessment of tumor characteristics than histopathological grading and could serve as a complementary approach to invasive tissue sampling in the future. Structured MRI datasets can be utilized both for systematic analyses and as training sets for AI applications. While AI requires large datasets, it has the potential to deliver rapid, image-based diagnostic assessments, which could reduce diagnostic delays and accelerate treatment initiation. Ultimately, standardized imaging enhances the reliability of treatment allocation and provides a foundation for ongoing diagnostic development. This is particularly crucial for rare tumor entities, such as soft tissue sarcomas, where such analyses are indispensable for advancing diagnostic standards.

Moreover, a comparable imaging protocol is essential not only for initial staging but also for the evaluation of treatment response and long-term follow-up. The German S3 guideline recommends regular MRI of the tumor region in combination with CT of the lungs for surveillance. A lack of standardization may significantly impede the accurate differentiation between post-therapeutic changes and tumor recurrence [38, 39]. 

Similar standardization challenges, as seen for STS, have also occurred in other tumor entities, such as rectal cancer. There, implementing a radial imaging plane for optimal mesorectal assessment required years of dissemination before becoming routine. Its benefits are now confirmed by prospective multicenter data and firmly established in the German S3 guideline, providing a model for accelerating the adoption of standardized imaging protocols in other entities such as STS [40, 41]. 

This retrospective study is based on a limited cohort. Regional differences – particularly with regard to reimbursement – cannot be taken into account due to the monocentric study design. While repeat outpatient imaging is routinely performed in some centers, this was not standard practice in the investigated center. Although this analysis was conducted on a small, limited cohort, the findings reveal a consistent trend. Expanding the evaluation to several hundred additional cases to achieve a significance case number would likely not have provided meaningful added value.

In summary, compliance with the S3 guideline depends on the precision of the clinical question, awareness of the guideline, and the financial feasibility of performing additional sequences and extended scan times. A potential measure for improvement would be the establishment of a clear, minimal protocol including T1- and T2-weighted sequences before and after contrast administration. Incorporating DWI as part of the standard protocol also appears useful for functional radiological analysis e.g. grading. The recommendations of the S3 guideline should be re-evaluated, which could be undertaken in the context of the planned update of the guideline from 2026 onwards. Furthermore, awareness of the diagnosis of rare STS should be strengthened among both referring physicians and radiologists. Close interdisciplinary collaboration between radiology and surgery may further enhance imaging for surgical planning. Clear open-access publication of standard imaging protocols would be a valuable contribution in this regard.

## Conclusion

This study aimed to evaluate compliance with the German S3-guideline for magnetic resonance imaging in the pretherapeutic staging of adult STS in routine clinical practice. Our analysis reveals that full adherence to the recommended comprehensive MRI protocol - especially in non-specialized, non-academic outpatient centers - is achieved in only a small fraction of cases, mainly due to limited guideline awareness, economic constraints, and variable clinical indications. Despite incomplete protocol adherence, current imaging practices generally allow for adequate anatomical assessment to guide surgical and therapeutic decisions. However, to enable precise tumor characterization, optimized surgical planning, improved prognostic accuracy, and enhanced multicenter research and artificial intelligence applications, standardized and guideline-conform MRI protocols - including functional sequences such as DWI - are essential. Future efforts should focus on increasing awareness, simplifying protocol dissemination, and integrating novel imaging acceleration technologies to reconcile high diagnostic quality with practical feasibility. These steps are poised to improve outcome-based therapy planning and ultimately patient care in this rare and challenging disease entity.

## Data Availability

The datasets used and/or analyzed during the current study are available from the corresponding author on reasonable request.

## Abbreviations

ADC: Apparent diffusion coefficient
AI: Artificial intelligence
AWMF: Arbeitsgemeinschaft der Wissenschaftlichen Medizinischen Fachgesellschaften (Translation: Working Group of the Scientific Medical Societies)
CT: Computed tomography
DCE: Dynamic contrast‑enhanced
DWI: Diffusion-weighted imaging
EBM: Uniform value scale for reimbursement
ESSR: European Society of Musculoskeletal Radiology
FNCLCC: Fédération Nationale des Centres de Lutte Contre le Cancer
G: Grade
K_ep_: rate constant
K_trans_: volume transfer constant
MRI: Magnetic resonance imaging
PWI: Perfusion‑weighted imaging
SE: Spin echo
STS: Soft tissue sarcoma
SWI: Susceptibility‑weighted imaging
TIC: Time-intensity curve
TSE: Turbo spin echo
